# Epidémiologie et prise en charge de l'insuffisance cardiaque dans un centre marocain

**DOI:** 10.11604/pamj.2016.24.85.8521

**Published:** 2016-05-27

**Authors:** Jamal Kheyi, Abdelilah Benelmakki, Hicham Bouzelmat, Ali Chaib

**Affiliations:** 1 Service des Soins Intensifs et Rythmologie, Hôpital Militaire d'Instruction Mohammed V, Rabat, Maroc

**Keywords:** Insuffisance cardiaque, épidémiologie, étiologie, traitement, Maroc, Heart failure, epidemiology, etiology, treatment, Morocco

## Abstract

Il s'agit d'une étude observationnelle prospective colligée dans le service des soins intensifs et rythmologie de l'Hôpital militaire d'instruction Mohammed V de Rabat, entre décembre 2008 et décembre 2014, portant sur 424 patients hospitalisés pour insuffisance cardiaque. L’âge moyen était de 60,91±12,77 Les principaux facteurs de risque cardiovasculaires rencontrés étaient: l'hypertension artérielle (46%), le tabagisme (45%), et le diabète (43%). Cliniquement, 63% des patients ont été admis pour insuffisance cardiaque gauche. Le ventricule gauche était dilaté dans 58% des cas, avec une fraction d’éjection moyenne estimée à 36,33±13,5%. L’étiologie dominante était la cardiopathie ischémique (254 cas). En plus d'un traitement médical optimal, 14.4% de nos patients ont fait l'objet d'une resynchronisation cardiaque avec ou sans système de défibrillation. L’évolution intra-hospitalière sous traitement médical était marquée par 26 décès.la durée moyenne d'hospitalisation était de 12,1±6,6 jours.

## Abstract (en)

This is a prospective observational study of 424 patients hospitalized for heart failure. Data were collected from the intensive care unit and Rhythmology Depatment of the Mohamed V Military Hospital for Instruction in Rabat, between December 2008 and December 2014. The average age was 60.91±12.77. The main cardiovascular risk factors encountered were: hypertension (46%), smoking (45%), and diabetes (43%). Clinically, 63% of patients were admitted for left-sided heart failure. The left ventricle was dilated in 58% of cases, with a mean ejection fraction estimated at 36.33 ± 13.5%. The dominant etiology was ischemic heart disease (254 cases). In addition to optimal medical treatment, 14.4% of our patients underwent cardiac resynchronization with or without defibrillator system. The intra-hospital evolution under medical treatment was marked by 26 deaths. The average length of stay in hospital was 12.1 ± 6.6 days.

## Introduction

L'insuffisance cardiaque (IC) est une pathologie fréquente et grave. Son épidémiologie est peu connue dans notre pays malgré son impact économique considérable sur le système de santé. Sa prévalence ne cesse d'augmenter du fait du vieillissement de la population et du meilleur pronostic des maladies qui conduisent à l'insuffisance cardiaque, notamment la maladie coronaire et l'hypertension artérielle.

## Méthodes

**Présentation de l’étude:** Nous avons mené une étude observationnelle prospective colligée dans le service des soins intensifs et rythmologie de l'hôpital militaire d'instruction Mohammed V de Rabat, entre décembre 2008 et décembre 2014, portant sur 424 patients

**Critères d'inclusion:** Ont été inclus dans l’étude tous les patients âgés de plus de 18 ans et hospitalisés dans le service des soins intensifs pour des signes cliniques et/ou échocardiographiques d'insuffisance cardiaque.

**Recueil des données:** Les paramètres analysés ont été les suivants: données épidémiologiques, présentation clinique de l'insuffisance cardiaque, anomalies électrocardiographiques, radiologiques et échocardiographiques, nature de la cardiopathie sous jacente, modalités thérapeutiques et évolution en cours d'hospitalisation sous traitement conventionnel.

**Analyse statistique:** L'analyse statistique a été réalisée à l'aide d'un logiciel SPSS version 13.0. Les variables quantitatives ont été exprimées en moyenne plus ou moins écart-type. Les variables qualitatives ont été exprimées en effectif et pourcentage.

## Résultats

**Figure 1:**
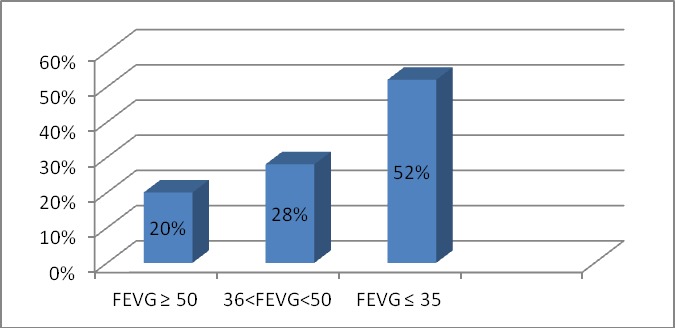
Répartition en fonction de la FEVG.

Durant la période étudiée, 424 patients ont été admis pour insuffisance cardiaque. L’âge moyen de nos patients était de 60,91±12,77 ans, en majorité des hommes (72%), avec un sexe ratio à 2,5. 90 patients avaient un antécédent d’événement cardiovasculaire. Les principaux facteurs de risque cardiovasculaire rencontrés étaient: l'hypertension artérielle (46%), le tabagisme (45%), le diabète (43%), la dyslipidémie (26%) et le surpoids (22%). Cliniquement, 63% des patients ont été admis pour insuffisance cardiaque gauche, 29% pour insuffisance cardiaque globale et 8% pour insuffisance cardiaque droite. 54% de nos patients étaient en insuffisance cardiaque stade III-IV, et 46% en stade I-II de la NYHA. La fréquence cardiaque moyenne était de 98±21 bpm. 65% de nos patients avaient une fréquence cardiaque ≥80cpm. Tous nos patients ont fait l'objet d'un électrocardiogramme, le Tableau 1 résume les principales données recueillies. A la radiographie pulmonaire, la cardiomégalie était présente dans 86% des cas, avec des signes de surcharge pulmonaire dans 82% des cas. 23% de nos patients étaient en insuffisance rénale modérée à sévère, 21% en anémie,6% en hyponatrémie et 4% en hypokaliémie. L’échocardiographie transthoracique retrouvait un ventricule gauche (VG) dilaté dans 58% des cas, avec un diamètre télédiastolique moyen à 60±9,6 mm, un diamètre télésystolique moyen à 45±12,5mm et une fraction d’éjection (FE) moyenne à du VG à 36,33±13.5%. Figure 1. Les pressions de remplissage du VG étaient élevées dans 60% des des cas, et l'hypertension artérielle pulmonaire était présente dans 43% des cas. La coronarographie réalisée chez 343 patients a montré un réseau coronaire normal dans 30% des cas, une atteinte monotronculaire dans 25,60% des cas, une atteinte tritronculaire dans 24,7% des cas, une atteinte bitronculaire dans 14,5% des cas et une sténose significative du tronc commun gauche dans 5,2% des cas. Les étiologies sont dominées par la cardiopathie ischémique (254 cas), la cardiomyopathie dilatée (CMD) d'allure primitive (69 cas), les valvulopathies (54 cas), la CMD secondaire à une dysthyroidie (10 cas), la non-compaction du VG (4 cas), le cœur pulmonaire chronique (CPC) (3 cas), la péricardite chronique constrictive (PCC) (3cas) et d'autres étiologies (27 cas). Le Tableau 2 résume les pourcentages des différentes étiologies. Le Tableau 3 représente les principales causes de décompensation rencontrées. A l'entrée, nos patients ont été mis sous traitement médical composé de: Furosémide (99%), inhibiteurs de l'enzyme de conversion (IEC) (68%), spironolactone (29%), bétabloquants (18%), antagonistes de récepteurs de l'angiotensine II (ARA II) (7,5%) et inotropes positifs (7,8%). 21,3% des patients ont fait l'objet d'une angioplastie coronaire transluminale, 17,3% d'un pontage aorto-coronaire, 14,4% d'une resynchronisation cardiaque dont 16 avec système de défibrillation et 8,8% d'une chirurgie valvulaire. Le traitement de sortie était composé de: Furosémide (54%), IEC (81%), bétabloquants (76%), spironolactone (58%) et ARA II (12%). 81 patients ont été hospitalisés pour plusieurs poussées d'IC par an. Le taux de décès en intrahospitalier était de 6,1%, en majorité des hommes âgés de plus de 55ans. La durée moyenne de l'hospitalisation était de 12,1±6,6 jours.

**Table 1:** Principales données électrocardiographiques recueillies

Anomalies électriques	Pourcentage (%)
Onde Q de nécrose	33
Bloc de branche gauche	31
Hypertrophie ventriculaire gauche	29
Troubles de la repolarisation	26
Fibrillation atriale	21

**Table 2:** Causes de l'insuffisance cardiaque

Cause	Pourcentage (%)
Cardiopathie ischémique	59,91
CMD d'allure primitive	16,27
Valvulopathie	12,74
Dysthyroidie	2,36
Non compaction du VG	0,94
CPC	0,71
PCC	0,71
Autre	6,36

**Table 3:** Causes de la décompensation de l'IC

Cause de décompensation	Nombre
Poussée ischémique	170
Surinfection bronchique	87
Ecart de régime	79
Trouble de rythme	48
Arrêt du traitement	11

## Discussion

Le nombre de patients insuffisants cardiaque est en augmentation constante dans le monde. Le vieillissement de la population et l'amélioration de la prise en charge des coronaropathies et de l'hypertension artérielle qui restent les principales étiologies [1]. En occident, la prévalence de l'insuffisance cardiaque est connue. Tel n'est pas le cas en Afrique [2] notamment en Afrique du nord. Les données de la société européenne de cardiologie suggèrent qu'il y a moins de 15 millions de patients souffrant d'insuffisance cardiaque en Europe, sur une population de 900 millions d'habitants répartis dans 51 pays [1]. Quant aux Etats- unis, il y a approximativement 5 millions d'américains souffrant d'insuffisance cardiaque, et plus de 550000 nouveaux cas sont diagnostiqués chaque année [3]. Nous avons comparé le profil de nos patients insuffisants cardiaques à celui identifié par une étude casablancaise (profil épidémiologique, prise en charge et évolution de 1578 patients avec insuffisance cardiaque chronique) [4], par une étude sub-saharienne (insuffisance du sujet âgé à Brazzaville) [2] et par d'autres grandes études telles que Acute Decompensated Heart Failure Survey National Registery (ADHERE) [5], EURO Heart Failure Survey (EHFS I) [6], Euro Heart Failure survey II (EHFS II) [7] Observatoire français de l´insuffisance cardiaque aiguë (OFICA) [8], Observatoire permanent de l´insuffisance cardiaque (ODIN) [9], Heart Failure Pilot Survey (ESC-HF Pilot) [10] et Thai Acute Decompensated Heart Failure Registry (Thai ADHERE) [11]. L’âge moyen des insuffisants cardiaques varie selon les différentes études entre 64 et 77 ans. Nos patients sont les plus jeunes comparativement aux américains, européens et thaïlandais. La majorité de nos patients sont de sexe masculin ce qui rejoint les données de la littérature. L'insuffisance cardiaque gauche est la présentation clinique de loin la plus fréquente dans notre étude. En afrique sub-saharienne, c'est l'insufisance cardiaque globale qui prédomine, et il s'agit d'une forme habituelle, comme l'ont relevé la plupart des auteurs. L'hypertrophie ventriculaire gauche assez fréquente chez nos patients est un puissant et indépendant facteur de risque cardio-vasculaire et multiplie à elle seule le risque de l'insuffisance cardiaque par 15. L'ETT a été réalisée chez tous nos patients, car elle constitue un examen-clé dans la prise en charge de l´insuffisant cardiaque, que ce soit au moment du diagnostic de la maladie ou au cours du suivi. L'origine de l'insuffisance cardiaque est surtout ischémique dans notre série et dans les études européennes, américaines et thaïlandaises. Au Congo [2], c´est l'hypertension artérielle qui est la principale cause de l'insuffisance cardiaque chez l'adulte africain et constitue un véritable problème de santé publique La prévalence de la maladie coronaire n´est pas bien définie et est certainement sous-estimée. L´augmentation de la cardiopathie ischémique dans notre pays s´explique par les moyens diagnostiques plus performants, mais peut-être aussi par le changement du mode de vie et l´urbanisation.

La pathologie valvulaire représentait 12,74% des causes d´insuffisance cardiaque dans notre étude et de l'ordre de 9,4% dans l’étude sub-saharienne. Elle était dominée par les atteintes mitro-aortiques. Si dans les pays développés leur mécanisme est souvent dystrophique ou dégénératif, dans les pays en développement, par contre, il s´agit encore et souvent d´atteintes rhumatismales survenues pendant le jeune âge et évoluant spontanément en l'absence de possibilités de chirurgie cardiaque ou de cardiologie interventionnelle. Des études américaines et européennes montrent parfaitement que les recommandations pour l'insuffisance cardiaque en matière de traitement sont trop peu appliquées. Le sous-traitement par IEC, bêta-bloquants et spironolactone est énorme. Les patients en insuffisance cardiaque à haut risque surtout, par exemple ceux en insuffisance rénale en plus, ne bénéficient souvent d´aucun traitement par bêta-bloquants et/ou IEC, alors que ce sont eux qui en profiteraient le plus. Plusieurs études ont montré que la mise en pratique de ces recommandations est nettement meilleure dans le cadre d'une prise en charge en réseau. La compliance des patients, surtout, peut s´en trouver améliorée, pas seulement pour le traitement, mais aussi pour l'autocontrôle et le respect des mesures d´accompagnement, ce qui non seulement améliore leur qualité de vie mais encore diminue les hospitalisations et même la mortalité. Pour nos patients, le traitement médical était maximal chaque fois que l’état hémodynamique le permettait chez 220 d´entre eux, soit 52%. Chez les patients atteints d'insuffisance cardiaque chronique et qui malgré un traitement médicamenteux optimisé restent symptomatiques, un asynchronisme interventriculaire est fréquemment retrouvé. Cet asynchronisme peut de nos jours être facilement diagnostiqué et soigné par un traitement de resynchronisation cardiaque qui permet un bénéfice clinique significatif, un remodelage inverse avec réduction des volumes cardiaques et une baisse de la morbi-mortalité chez les patients insuffisants cardiaques symptomatiques avec QRS large [12]. De ce fait et au vu de l´orientation rythmologique de notre service, 14,4% de nos patients ont bénéficié d´une resynchronisation cardiaque avec ou sans système de défibrillation. Le pronostic de cette pathologie reste sombre, et il faut signaler que sa mortalité est plus importante que celles de l'infarctus du myocarde et de plusieurs cancers. Dans notre étude, le taux de décès était de 6,1%, d´où la nécessité d´une prise en charge spécifique adaptée et précoce.

## Conclusion

L'insuffisance cardiaque est un problème majeur de santé publique, et il l'est de plus en plus. La cardiopathie ischémique est la principale étiologie. La fréquence et la gravité de cette maladie doivent nous inciter à traiter nos patients au mieux, en utilisant nos ressources au maximum et en expliquant en détail aux patients le bien-fondé de la thérapeutique, y compris diététique. Cela doit également nous inciter à développer des structures de prise en charge telles qu'un hôpital de jour ou de consultations spécialisées pour améliorer le pronostic de cette lourde pathologie.

### Etat des connaissances actuelle sur le sujet

- Les valvulopathies rhumatismales étaient la principale étiologie au Maroc.
- Le pronostic est défavorable.
- Le cout élevé de la prise en charge.

### Contribution de notre étude à la connaissance

- Changement épidémiologique avec augmentation de la fréquence des coronaropathies.
- Age jeune de nos patients.
- Nécessité d'instaurer des unités thérapeutiques d'insuffisance cardiaque.
